# Measles Seroprevalence Among Healthcare Workers in a Tertiary Hospital in Central Greece, 2017

**DOI:** 10.3390/vaccines14050379

**Published:** 2026-04-23

**Authors:** Eirini Karnava, Marios Politis, Efthymia Petinaki, Konstantinos I. Gourgoulianis, Christos Hadjichristodoulou, Georgios Rachiotis

**Affiliations:** 1 Department of Hygiene and Epidemiology, School of Medicine, University of Thessaly, 41222 Larissa, Greece; 2 Department of Medical Biopathology, University Hospital of Larissa, University of Thessaly, 41334 Larissa, Greece; 3 Department of Respiratory Medicine, Faculty of Medicine, School of Health Sciences, University of Thessaly, 41110 Larissa, Greece

**Keywords:** healthcare workers, measles, seroprevalence study, cross-sectional study

## Abstract

Background: Measles remains a significant occupational hazard in healthcare settings. In the context of the 2017–2018 measles outbreak in Greece and amid an outbreak at the study hospital, this seroprevalence study aimed to identify gaps in measles serologic status among healthcare workers in a tertiary hospital in central Greece. Methods: We conducted a seroprevalence study among hospital employees between February and December 2017. Blood samples and data on sociodemographic and work-related characteristics were collected from a convenience sample of participants. Measles IgG and IgM antibodies were measured using the ELISA method to determine seropositivity. The 95% CIs for measles IgG seronegativity proportions were calculated using the Clopper–Pearson exact method. Associations between participant characteristics and measles antibody status were assessed using Firth’s penalized logistic regression models. Results: A total of 336 healthcare workers participated in the study (response rate: 24.9%). Overall, 5.4% (95% CI: 3.2–8.3) tested negative for measles IgG antibodies. No significant associations were observed between participants’ characteristics and measles IgG antibody status. Male participants had 15.8 times higher adjusted odds of testing positive for measles IgM antibodies compared with female participants (aOR: 15.8; 95% CI: 2.33–107.54; *p* = 0.005). Conclusions: Our results indicate a low—but not negligible—proportion of IgG measles seronegativity among participants. The detection of seronegative individuals born prior to 1970 challenges the assumption of universal natural immunity based solely on year of birth. Given the recent rise in measles outbreaks and the limited seroprevalence data among healthcare workers in Greece, these findings provide valuable data to support ongoing efforts to achieve full vaccination coverage in this group. Further research is warranted to investigate the observed sex differences in susceptibility to measles infection.

## 1. Introduction

Measles, a highly contagious respiratory viral disease, caused approximately 95,000 deaths globally in 2024 and remains a leading cause of mortality among children under five [1]. The introduction of measles vaccination in 1968 led to a substantial global decline in incidence, with 82 countries achieving disease elimination by 2018, representing progress toward the World Health Organization (WHO) Measles and Rubella Strategic Framework (MRSF) 2021–2030 [2,3]. Despite these gains, measles continues to cause outbreaks worldwide, primarily among under-vaccinated populations [4]. In countries with adequate measles vaccination coverage, outbreaks in healthcare settings are of greatest concern, with transmission often occurring between healthcare workers (HCWs) and patients [5,6].

Recognizing the importance of vaccination among HCWs, the WHO recommends measles vaccination for this group, and professional societies such as the American College of Preventive Medicine (ACPM) support mandatory vaccination for this population [7,8]. However, national adoption of HCW measles vaccination policies has been inconsistent, with only about half of European countries implementing such policies [9]. Reflecting this variability, a systematic review of 98 studies reported global measles seropositivity among HCWs ranging widely from 57.1% to 99.2% [10]. In Greece, measles seroprevalence data among HCWs are very limited, with two studies conducted in 2009 reporting seropositivity rates of 97.9% and 98.4% among HCWs [9]. These estimates predate the recent resurgence of measles in Europe and may not adequately represent current immunity levels among HCWs in the context of recent epidemiological shifts.

After a long decline between 2000 and 2016, global measles cases increased in 2018, reaching the highest level since 2011 [11,12]. Europe reported over 80,000 cases that year, with Greece recording 3150 cases in 2017–2018 [13,14]. These outbreaks were attributed to suboptimal vaccination coverage and international travel, with imported cases spreading predominantly among under-immunized populations [12,14]. Several outbreaks involved HCWs, with Italy and Greece accounting for the highest numbers, including 65 HCWs infected in Greece [15]. Recent surveillance data from the European Centre for Disease Prevention and Control (ECDC) indicate a nearly ten-fold increase in measles cases in the EU/EEA in 2024 compared with 2023, highlighting a marked resurgence of transmission across Europe [16].

Following the measles outbreak in Greece in 2017–2018, and during a concurrent outbreak at the study hospital, the aim of our study was to identify gaps in measles serologic status among HCWs employed at this tertiary hospital in central Greece and to explore potential associations between seroprevalence and various sociodemographic and work-related characteristics. The study addressed the following research questions:

1. What proportion of HCWs were tested negative for measles IgG antibodies?
2. Are there statistically significant associations between HCWs’ measles IgG antibody status and common sociodemographic or work-related characteristics, and if so, what is the direction and strength of these associations?
3. Are there statistically significant associations between HCWs’ measles IgM antibody status and common sociodemographic or work-related characteristics, and if so, what is the direction and strength of these associations?

The persistent risk of measles transmission in healthcare facilities, combined with the very limited recent seroprevalence data from Greece, creates an important evidence gap for informing occupational and public health policy and outbreak preparedness. By addressing the above-mentioned research questions, the study aims to provide contemporary evidence to inform occupational and public health policies and outbreak preparedness in Greek and similar European healthcare settings.

## 2. Materials and Methods

### 2.1. Study Reporting

This study was reported in accordance with the STROBE (Strengthening the Reporting of Observational Studies in Epidemiology) guidelines.

### 2.2. Sample and Study Participants

Our study sample was based on a convenience sample. Between February and December 2017, all HCWs at the University Hospital of Larissa, Greece, were invited to participate through the occupational physician. HCWs involved in patient care were eligible for inclusion in the study. During the study period, 1352 HCWs were actively working at the hospital. In total, 336 participants were included in the study, yielding a response rate of 24.9%.

### 2.3. Blood Sample Collection and Serum Samples Analysis

Blood samples were collected at the hospital’s occupational medicine department by clinic personnel and transported to the hospital’s virology laboratory on the same day. A quantitative enzyme-linked immunosorbent assay (ELISA) was used to detect measles-specific IgG and IgM antibodies in the participants’ serum samples. For the detection of IgG antibodies, the Virion/Serion ELISA kit (Institut Virion\Serion GmbH, Würzburg, Germany) was used, with a reported sensitivity of >99% and a specificity of 95.0%, according to the manufacturer. The Virion/Serion ELISA kit (Germany) was also used for IgM antibodies, with the manufacturer reporting a sensitivity of 98.9% and a specificity exceeding 99%.

### 2.4. Informed Consent and Ethical Approval

Participation in the study was voluntary, with no monetary incentives offered, and all participants were asked to provide informed consent. Due to the public health emergency caused by the measles outbreak, obtaining written informed consent from all participants was not practical or feasible; therefore, verbal informed consent was obtained. All hospital workers were called in to be examined by the hospital’s occupational physician due to the ongoing outbreak. During these visits, verbal informed consent was documented in the patient/participant journal by the occupational physician. The study protocol was approved by the Steering Committee of the Postgraduate Program “Primary Health Care” at the Medical Faculty, University of Thessaly, Hellenic Republic (No. 57/20.02.2019). This approach aligns with established international guidelines for public health emergencies and outbreak responses (American College of Epidemiologists, Ethics Guidelines 2.6.3; International Ethical Guidelines for Health-related Research Involving Humans, Guideline 10) [17,18].

### 2.5. Measles Antibodies Status

In accordance with the manufacturer’s guidelines, individuals with IgG antibody titers greater than 200 mIU/mL were considered positive, as measles IgG concentrations above this threshold are recognized as indicative of seroprotection against measles infection—a cutoff that is also widely used in other measles seroprevalence studies [10,19]. The cut-off for measles IgM seronegativity was defined as <15 mIU/mL, indicating a non-recent measles infection.

### 2.6. Sociodemographic and Working-Related Characteristics

All participants were required to provide information about their sociodemographic and work-related characteristics. They were asked to specify their sex, age, department of service, and years of employment in healthcare sector. Regarding the service departments, the medical department comprised consultants and residents, the nursing department comprised nursing staff, and the paramedical department included patient transport staff, auxiliary nursing personnel, and technicians (e.g., radiology equipment technicians).

### 2.7. Statistical Analysis

All statistical analyses were performed using Stata version 18 (StataCorp LLC, College Station, TX, USA). Categorical variables are presented as counts and corresponding percentages, and continuous variables as medians with interquartile ranges (IQRs). The 95% confidence intervals (95% CIs) for IgG seronegativity proportions were calculated using the Clopper–Pearson exact method.

Given the small number of cases for both outcomes (n = 18 and n = 6), Firth’s penalized likelihood logistic regression was used to reduce small-sample bias and prevent separation. Associations between sociodemographic and work-related characteristics and measles IgG and IgM antibody status were assessed using both univariable and multivariable models. To preserve statistical power and minimize overfitting, age was included as a continuous variable in the models. Variables included in the multivariable logistic regression model to estimate adjusted odds ratios (aOR) were sex, age, years of employment, and department. Statistical significance was defined as *p* < 0.05, and 95% CIs were reported for all estimates.

## 3. Results

Table 1 presents the sociodemographic and work-related characteristics, as well as measles IgG antibody status, of the study participants. A total of 336 participants were included in the study, with a median age of 46 years (IQR: 41–49). Female participants and those working in the nursing department accounted for 79.5% and 73.8% of the sample, respectively. The median years of employment was 12 (IQR: 1–18), with half of the participants having between 0 and 10 years of work experience.

Table 2 shows that the overall prevalence of measles IgG seronegativity among participants was 5.4% (95% CI: 3.2–8.3). In total, 18 individuals tested negative for measles IgG antibodies (see Appendix A Table A1). Higher IgG seronegativity rates were observed among males (8.7%; 95% CI: 3.3–18.0) and medical staff (11.8%; 95% CI: 3.3–27.5), whereas lower proportions were found among paramedical personnel (1.9%; 95% CI: 0.0–10.0) and employees with more than 20 years of service (3.7%; 95% CI: 0.4–12.7). IgG seronegativity estimates were similar across age groups. The substantial overlap of 95% CIs across sex, age, and occupation groups suggests homogeneous measles seronegativity among the participants.

Table 3 shows the univariable and multivariable associations between sociodemographic and work-related characteristics and measles IgG antibody status, presented as crude and adjusted ORs. Although a tendency was observed for female participants (aOR = 1.83; 95% CI: 0.62–5.41; *p* = 0.27), as well as for nursing (aOR = 1.82; 95% CI: 0.52–6.35; *p* = 0.34) and paramedical staff (aOR = 3.83; 95% CI: 0.51–28.73; *p* = 0.19) compared to medical staff, to have higher odds of IgG measles antibody seropositivity, no statistically significant associations were found.

Table 4 presents the associations between sociodemographic and work-related characteristics and measles IgM antibody status among participants. In total, 6 individuals tested positive for measles IgM antibodies (see Appendix A Table A1). Male participants had significantly higher odds of IgM seropositivity compared with females in both the univariable (OR = 15.15; 95% CI: 2.44–94.04; *p* = 0.04) and multivariable analyses (aOR = 15.80; 95% CI: 2.33–107.54; *p* = 0.005). In contrast, age and years of employment were not significantly associated with IgM antibody status. Although medical and nursing staff showed higher odds of IgM seropositivity than paramedical staff, these differences did not reach statistical significance.

## 4. Discussion

In this study, we assessed potential gaps in measles serologic status among HCWs employed at a tertiary hospital in central Greece between February and December 2017 and examined associations between measles IgG and IgM antibody status and selected sociodemographic and work-related characteristics. A total of 336 HCWs participated (response rate: 24.9%), with 5.4% found to be seronegative for measles IgG antibodies. No statistically significant associations were observed between IgG serologic status and any sociodemographic or work-related variables. In the context of the outbreak investigation, six participants, all of whom were symptomatic, tested positive for IgM measles antibodies, indicating a recent measles infection. When exploring factors associated with confirmed recent measles infection, a significant difference by sex was identified, with male participants showing higher odds of measles IgM positivity compared with females (aOR: 15.8; 95% CI: 2.33–107.54; *p* = 0.005).

In the context of this particular outbreak investigation, preventive measures for suspected or confirmed measles cases among HCWs included exclusion from work for at least four days following rash onset. Among unimmunized HCWs—given that vaccination is not mandatory in the Greek healthcare context—active surveillance with daily symptom monitoring was implemented, while measles–mumps–rubella (MMR) vaccination was strongly recommended. Additionally, all HCWs were strongly advised to adhere to infection prevention measures, including the use of surgical masks and regular hand hygiene.

A 2022 meta-analysis of 19 studies from European countries, which did not include data from Greece, reported a pooled measles IgG seronegativity of 13.3% (95% CI: 10.0–17.0%) among HCWs, which is 7.9 percentage points higher than the estimate observed in our study [20]. Similarly, a systematic review published in 2014 reported a median measles IgG seronegativity rate of 6% in Europe (range: 0.9–16.9%) and included two studies conducted in Greece in 2009, which reported IgG seronegativity rates of 2.1% and 1.6% among HCWs [9]. However, these studies involved populations that differed substantially from ours, with one study including medical and nursing students with a median age of 23 years, while the other included hospital staff aged 28–50 years [9].

More recently, a systematic review published in 2025 reported measles IgG seronegativity rates across European countries ranging from 2% in Spain to 33.3% in Italy but did not include any studies from Greece [10]. The variation in IgG seronegativity estimates across studies included in these reviews likely reflects heterogeneity in study populations, laboratory methods, and seropositivity thresholds, as well as differences in study design and sampling approaches. Notably, the inclusion of only two studies from Greece—both published in 2009—in all three relevant systematic reviews underscores the scarcity of data on measles seroprevalence among Greek HCWs.

In our study, no significant differences in measles IgG seronegativity were observed across sociodemographic or occupational characteristics, suggesting relatively homogeneous levels of serologic status among hospital staff. This finding aligns with evidence showing similar measles seroprevalence among HCWs across different health professions, sexes, and with increasing age [21,22]. Although other studies have also reported no difference in measles IgG serologic status with increasing age, it is well documented that younger individuals born after 1980 tend to exhibit higher rates of IgG seronegativity, which may reflect waning immunity in populations that rely solely on vaccine-induced protection without natural exposure to measles [10,23].

Our multivariable analysis indicated that male participants were significantly more likely to test positive for a recent measles infection. This is consistent with evidence showing higher measles incidence in males, particularly younger individuals, across regions and over time, with explanations favoring physiological and biological mechanisms over behavioral factors [24]. Measles antibody responses to infection or vaccination differ by sex, with females generally exhibiting higher titters, particularly among those aged 60 years and older [25,26,27]. In our study, all participants who were IgM-positive were also IgG-negative, indicating no evidence of prior immunity among these individuals. In the absence of data on vaccination status, this finding may reflect either infection in an unvaccinated individual or infection in an individual with vaccine failure, which has been reported in approximately 2–10% of measles-vaccinated individuals [28]. However, it should be noted that, based on current serological cut-offs, a recent meta-analysis found that sex was not a significant source of heterogeneity in measles seropositivity among previously vaccinated individuals [29]. Behavioral and cultural factors may further contribute to these sex differences, as males often engage in riskier behaviors compared to females [30,31].

An interesting and clinically relevant finding of the present seroprevalence study was the presence of seven measles IgG seronegative HCWs born before 1970. According to the Greek National Immunization Program, individuals born before 1970 are generally presumed to be immune to measles due to the widespread natural circulation of the wild-type virus prior to the introduction of measles vaccination in the mid-1970 [32]. However, several studies have shown that 1–2% of people born before 1970 remain seronegative for measles in both HCWs and the general population [9,33]. The detection of seronegative individuals in this older birth cohort challenges the assumption of universal natural immunity based solely on year of birth. Several explanations may account for this observation. First, not all HCWs born before 1970 were necessarily exposed to wild-type measles during childhood, particularly those living in rural areas or with limited social contacts. Second, measles-specific antibodies acquired through natural infection can wane over decades, although this occurs less frequently than with vaccine-induced immunity. Third, laboratory-related factors such as the specific ELISA used and the chosen cut-off value (200 mIU/mL) may classify some individuals with low but potentially protective antibody titers as seronegative. This finding underscores the limitations of relying exclusively on age-based criteria for determining measles immunity in high-risk occupational groups.

It should be noted that measles, with an R_0_ (basic reproduction number) of 12–18, the highest known R_0_ among human infectious diseases, requires a herd immunity level corresponding to seronegativity below 5% to prevent outbreaks [34]. Consequently, the interplay of measles seronegativity among the general population and HCWs plays a crucial role in measles outbreak prevention. A recent nationwide study from Greece reported a seronegativity rate of 11.4% in the general population during 2020–2021, highlighting a gap that could allow for potential measles outbreaks [35].

### 4.1. Strengths and Limitations

Our study provides timely, policy-relevant data on measles serologic status gaps among HCWs amid the resurgence of measles in Europe. Seroprevalence data from Greece are very limited, and our study helps address this gap with results from a relatively large sample of HCWs (n = 336). With measles cases in the EU/EEA rising ten-fold in 2024 compared with 2023, these findings are particularly relevant [16]. Moreover, we employed ELISA, a highly sensitive and specific method, to minimize the risk of misclassification, and applied Firth’s penalized logistic regression to appropriately analyse rare-event outcomes, yielding unbiased estimates.

A key limitation of this study is the use of a convenience sampling approach combined with a relatively low response rate, which may have introduced selection bias and limited the generalizability of our findings. Unfortunately, we lack data on non-responders, which prevents us from identifying and quantifying the potential for selection bias in our study. The underrepresentation of male participants may have further affected internal validity. In addition, we lacked information on documented vaccination status or prior measles exposure, which could have helped explain differences in IgG seronegativity. With only 18 participants testing IgG seronegative, our model may have been underpowered to detect modest effects, increasing the risk of type II error. For the IgM analysis, although we used methods appropriate for rare outcomes, the very low prevalence (n = 6) and the wide 95% CI reported mean that these findings should be considered exploratory and interpreted with caution.

### 4.2. Future Research Directions

Larger, multicenter seroprevalence studies across Greece are needed, ideally incorporating vaccination records and detailed occupational exposure data. Longitudinal studies assessing the durability of vaccine-induced immunity in HCWs and the effectiveness of different intervention strategies would provide important evidence for policymakers.

## 5. Conclusions

The present seroprevalence study among HCWs showed that a small but not negligible proportion (5.4%) tested negative for measles IgG antibodies. Given the high transmissibility of measles, even this level of susceptibility in a tertiary hospital poses a meaningful outbreak risk. Furthermore, according to the Greek National Immunization Program, individuals born before 1970 are generally presumed to be immune to measles. However, we found that a small proportion of individuals born before 1970 remained measles seronegative, challenging the assumption that this cohort has universal natural immunity. As measles outbreaks are resurging in Europe, these findings underscore the need for ongoing efforts to ensure full vaccination of all susceptible HCWs. Systematic documentation and verification of measles immunity should be prioritized, particularly in settings without mandatory vaccination policies. Last, further research is warranted to better understand the observed sex differences in susceptibility to measles infection.

## Figures and Tables

**Table 1:** Sociodemographic, work-related characteristics of the study participants (n = 336).

Characteristic n (%)	Total (N = 336)
**Sex**	
Male	69 (20.5)
Female	267 (79.5)
**Median Age (IQR), years**	46 (41–49)
**Age group**	
20–35 years	43 (12.8)
36–50 years	233 (69.3)
>50 years	60 (17.9)
**Service department**	
Medical	34 (10.1)
Nursing	248 (73.8)
Paramedical	54 (16.1)
**Years of employment, median (IQR)**	12 (1–18)
**Years of employment**	
0–10	158 (47.0)
11–20	124 (36.9)
>20	54 (16.1)

**Table 2:** Proportion (%) of measles IgG seronegativity among participants with corresponding 95% CIs.

Characteristic n (%)	Seronegativity—% (95% CI) (n = 18)
**Total**	5.4 (3.2–8.3)
**Sex**	
Male	8.7 (3.3–18.0)
Female	4.3 (2.3–7.4)
**Age group**	
20–35 years	6.9 (1.5–19.1)
36–50 years	5.1 (2.7–8.8)
>50 years	5.0 (1.0–13.9)
**Service department**	
Medical	11.8 (3.3–27.5)
Nursing	5.2 (2.8–8.8)
Paramedical	1.9 (0.0–10.0)
**Years of employment**	
0–10	6.3 (3.1–11.3)
11–20	4.8 (1.8–10.2)
>20	3.7 (0.4–12.7)

**Table 3:** Associations between sociodemographic and work-related characteristics and measles IgG antibody status (n = 336).

Characteristic	OR (95% CI)	*p*-Value	aOR (95% CI)	*p*-Value
**Sex**				
Male	Ref.		Ref.	
Female	2.09 (0.78–5.61)	0.14	1.83 (0.62–5.41)	0.27
**Age**	1.03 (0.97–1.10)	0.31	1.01 (0.94–1.08)	0.75
**Service department**				
Medical	Ref.		Ref.	
Nursing	2.57 (0.83–7.98)	0.14	1.82 (0.52–6.35)	0.34
Paramedical	5.26 (0.78–35.20)	0.09	3.83 (0.51–28.73)	0.19
**Years of employment**	1.03 (0.98–1.09)	0.21	1.01 (0.95–1.08)	0.71

**Table 4:** Associations between sociodemographic and work-related characteristics and measles IgM antibody status (n = 336).

Characteristic	OR (95% CI)	*p*-Value	aOR (95% CI)	*p*-Value
**Sex**				
Female	Ref.		Ref.	
Male	15.15 (2.44–94.04)	0.04	15.80 (2.33–107.54)	0.005
**Age**	0.97 (0.87–1.08)	0.35	0.99 (0.89–1.11)	0.92
**Service department**				
Paramedical	Ref.		Ref.	
Medical	8.38 (0.39–180.13)	0.17	5.27 (0.19–142.2)	0.32
Nursing	2.01 (0.11–37.08)	0.46	4.18 (0.19–89.67)	0.36
**Years of employment**	0.95 (0.86–1.05)	0.21	0.99 (0.90–1.10)	0.96

## Data Availability

The original contributions presented in this study are included in the article. Further inquiries can be directed to the corresponding author.

## Appendix A

**Table A1:** Absolute numbers and percentages of participants’ sociodemographic and work-related characteristics by measles IgG and IgM antibody status (n = 336).

Characteristic n (%)	IgG (+) (N = 318)	IgG (−) (N = 18)	IgM (+) (N = 6)	IgM (−) (N = 330)
**Sex**				
Male	63 (19.8)	6 (33.3)	5 (83.3)	64 (19.4)
Female	255 (80.2)	12 (66.7)	1 (16.7)	266 (80.6)
**Median Age (IQR), years**	46 (41–50)	44 (37–48)	44 (38–47)	46 (41–49)
**Service department**				
Medical	30 (9.4)	4 (22.2)	2 (33.3)	32 (9.7)
Nursing	235 (73.9)	13 (72.2)	4 (66.7)	244 (73.9)
Paramedical	53 (16.7)	1 (5.6)	0 (0.0)	54 (16.4)
**Years of employment, median (IQR)**	12 (1–18)	9 (1–12)	4 (1–8)	12 (1–18)

## References

[1] World Health Organization (2025). Measles—Fact Sheet.

[2] Pollard A.J., Bijker E.M. (2021). A guide to vaccinology: From basic principles to new developments. Nat. Rev. Immunol..

[3] World Health Organization (2020). Measles and Rubella Strategic Framework: 2021–2030.

[4] Centers for Disease Control and Prevention (2024). Global Measles Outbreaks—Data & Research.

[5] Botelho-Nevers E., Gautret P., Biellik R., Brouqui P. (2012). Nosocomial transmission of measles: An updated review. Vaccine.

[6] Heinze N., Martin A.F., Tirion A.S.C., Pae R., Islam J., Smith L.E., Weston D., Rubin G.J. (2025). Exploring the effectiveness of interventions to increase uptake of measles vaccination among healthcare workers: A systematic review. Vaccine.

[7] World Health Organization (2022). Implementation Guide for Vaccination of Health Workers.

[8] American College of Preventive Medicine (2025). Mandatory Vaccination for Healthcare Workers.

[9] Fiebelkorn A.P., Seward J.F., Orenstein W.A. (2014). A global perspective of vaccination of healthcare personnel against measles: Systematic review. Vaccine.

[10] Ng Y.F., Lockhart K., Dawar M., Vigor D., Yassi A. (2025). Assessing measles immunity among healthcare workers: A systematic review of peer-reviewed literature (2013–2023). Vaccine.

[11] Wang R., Jing W., Liu M., Liu J. (2022). Trends of the global, regional, and national incidence of measles, vaccine coverage, and risk factors in 204 countries from 1990 to 2019. Front. Med..

[12] Patel M.K., Dumolard L., Nedelec Y., Sodha S.V., Steulet C., Gacic-Dobo M., Kretsinger K., McFarland J., Rota P.A., Goodson J.L. (2019). Progress toward regional measles elimination—Worldwide, 2000–2018. MMWR Morb. Mortal. Wkly. Rep..

[13] Thornton J. (2019). Measles cases in Europe tripled from 2017 to 2018. BMJ.

[14] Georgakopoulou T., Horefti E., Vernardaki A., Pogka V., Gkolfinopoulou K., Triantafyllou E., Tsiodras S., Theodoridou M., Mentis A., Panagiotopoulos T. (2018). Ongoing measles outbreak in Greece related to the recent European-wide epidemic. Epidemiol. Infect..

[15] (2018). European Centre for Disease Prevention Control Risk of measles transmission in the, E.U./.E.E.A. https://www.ecdc.europa.eu/sites/default/files/documents/Measles-rapid-risk-assessment-European-Union-countries_0.pdf.

[16] European Centre for Disease Prevention and Control (2024). Measles Annual Epidemiological Report 2024. https://www.ecdc.europa.eu/sites/default/files/documents/MEAS-AER-2024-Report.pdf.

[17] American College of Epidemiology Ethics Guidelines. American College of Epidemiology. (n.d.). https://www.acepidemiology.org/ethics-guidelines.

[18] Council for International Organizations of Medical Sciences (CIOMS) (2016). International Ethical Guidelines for Health-related Research Involving Humans.

[19] Vittrup D.M., Jensen A., Malon M., Zimakoff A.C., Sørensen J.K., Littell B., Simões E.A., Svensson J., Stensballe L.G. (2024). Comparison of measles plaque reduction neutralization test (PRNT) and measles virus-specific IgG ELISA for assessment of immunogenicity of measles-mumps-rubella vaccination at 5–7 months of age and maternal measles antibodies. Vaccine X.

[20] Bianchi F.P., Stefanizzi P., Trerotoli P., Tafuri S. (2022). Sex and age as determinants of the seroprevalence of anti-measles IgG among European healthcare workers: A systematic review and meta-analysis. Vaccine.

[21] Lengyel G., Marossy A., Ánosi N., Farkas S.L., Kele B., Nemes-Nikodém É., Szentgyörgyi V., Kopcsó I., Mátyus M. (2019). Screening of more than 2000 Hungarian healthcare workers’ anti-measles antibody level: Results and possible population-level consequences. Epidemiol Infect..

[22] Urbiztondo L., Borràs E., Costa J., Broner S., Campins M., Bayas J.M., Esteve M., Domínguez A. (2013). Working Group for the Study of the Immune Status in Healthcare Workers in Catalonia. Prevalence of measles antibodies among health care workers in Catalonia (Spain) in the elimination era. BMC Infect. Dis..

[23] Anzà D., Restivo V., Cirillo F., Lo Sasso B., Martínez-Jarreta B., Senia P., Vitale E. (2025). Measles seroprevalence among health care professionals during a period of increased transmission: A pilot study in Southern Italy. IJID Reg..

[24] Green M.S., Schwartz N., Peer V. (2022). Gender differences in measles incidence rates in a multi-year, pooled analysis, based on national data from seven high income countries. BMC Infect. Dis..

[25] Flanagan K.L., Fink A.L., Plebanski M., Klein S.L. (2017). Sex and gender differences in the outcomes of vaccination over the life course. Annu. Rev. Cell Dev. Biol..

[26] Kostinov P.M., Zhuravlev I.P., Filatov N.N., Kostinova M.A., Polishchuk B.V., Shmitko D.A., Mashilov V.C., Vlasenko E.A., Ryzhov A.A., Kostinov M.A. (2021). Gender differences in the level of antibodies to measles virus in adults. Vaccines.

[27] Ruggieri A., Anticoli S., Di Ambrosio D., Giordani L., Viora M., Dipartimento D. (2016). The Influence of Sex and Gender on Immunity, Infection and Vaccination. http://www.iss.it/documents/20126/45616/ANN_16_02_11.pdf.

[28] Poland G.A., Jacobson R.M. (2012). The Re-Emergence of Measles in Developed Countries: Time to Develop the Next-Generation Measles Vaccines?. Vaccine.

[29] Mustafa M., Reznik A.S., Uribe J., Mintz J., Taldone S., Shukla B., Raja M., Natori Y. (2025). Measles seropositivity in previously vaccinated individuals: A systematic review and meta-analysis. EClinicalMedicine.

[30] van Lunzen J., Altfeld M. (2014). Sex differences in infectious diseases—Common but neglected. J. Infect. Dis..

[31] Klein S.L. (2000). The effects of hormones on sex differences in infection: From genes to behavior. Neurosci. Biobehav. Rev..

[32] Hellenic Republic Ministry of Health (2025). National Immunization Program for Adults 2025: Schedule and recommendations. National Immunization Program for Adults 2025.

[33] Springer D.N., Borsodi C., Camp J.V., Redlberger-Fritz M., Holzmann H., Kundi M., Aberle J.H., Stiasny K., Weseslindtner L. (2025). Seroprevalence against measles, Austria, stratified by birth years 1922 to 2024. Euro Surveill..

[34] World Health Organization Manual for the Laboratory-Based Surveillance of Measles and Rubella. Chapter 1.

[35] Nasika A., Bogogiannidou Z., Mouchtouri V.A., Dadouli K., Kyritsi M.A., Vontas A., Voulgaridi I., Tsinaris Z., Kola K., Matziri A. (2023). Measles immunity status of Greek population after the outbreak in 2017–2018: Results from a seroprevalence national survey. Vaccines.

